# Response of pet owners to Whistle FIT^®^ activity monitor digital alerts of increased pruritic activity in their dogs: a retrospective observational study

**DOI:** 10.3389/fvets.2023.1123266

**Published:** 2023-08-09

**Authors:** Aletha Carson, Cassie Kresnye, Taranpreet Rai, Kevin Wells, Andrea Wright, Andrew Hillier

**Affiliations:** ^1^Pet Insight Project, At-Home Diagnostics, Mars Science & Diagnostics, New York, NY, United States; ^2^The Veterinary Health Innovation Engine, School of Veterinary Medicine, University of Surrey, Guildford, United Kingdom; ^3^Zoetis, Parsippany, NJ, United States

**Keywords:** pruritus alert, scratching, licking, wearable activity monitor, dermatology, pet owner, deep learning computer algorithm, dog

## Abstract

Pruritus is a common clinical sign in dogs and is often underrecognized by dog owners and veterinarians. The Whistle FIT^®^, a wearable accelerometer paired with analytics, can detect changes in pruritic activity in dogs, which can be reported to owners in a smartphone/tablet application. The objectives of this retrospective observational study were to investigate the impact of digital alerts for increased pruritic behaviors received by dog owners in a real-life setting, on (1) the initiation of veterinary clinic visits, and (2) if such visits resulted in initiation of therapy for pruritus. Whistle FIT^®^ data and electronic health records from 1,042 Banfield veterinary clinics in the United States were obtained for a 20-month period and reviewed retrospectively. Data on times of increased pruritic behaviors was calculated retrospectively by the investigators by applying the same algorithms used in the Whistle system. Data from the first 10-month interval was compared to the second 10 months, when reports on pruritic behaviors and alerts for increased pruritic behaviors were viewable by pet owners. Signalment of dogs with clinic visits in the first (*n* = 7,191) and second (*n* = 6,684) 10-month groups was similar. The total number of pruritic alerts was 113,530 in the first 10 months and 93,217 in the second 10 months. The odds of an ‘alert visit’ (the first veterinary clinic visit that occurred within 4 weeks after the time of a pruritus alert) was statistically significantly more likely (odds ratio, 1.6264; 95% CI, 1.57–1.69; *p* < 0.0001) in the second 10-month period compared to the first 10-month period. The total number of medications administered was 10,829 in the first 10 months and 9,863 in the second 10 months. The percentage of medications prescribed within 4 weeks after a pruritus alert was higher in the second 10 month period (53.3%) compared to the first 10 month period (38.8%). This study suggests that pruritus alerts sent to dog owners may improve owner recognition of pruritic behaviors and increase the likelihood of a veterinary visit to treat canine pruritus.

## 1. Introduction

Pruritus is the most common clinical sign in small animal dermatology and may present without clinically evident skin disease (1). Canine pruritus may manifest as licking, scratching, chewing, rubbing, and overgrooming (2). It is estimated that up to 15% of medicalized dogs in the United States are affected by allergic skin disease or a dermatologic condition (3–5). Pet owners are often uncertain about what may be normal scratching behavior versus what is abnormal (6). An objective validated tool to assess normal versus excessive pruritic behavior is currently lacking. Canine pruritus has been shown to negatively impact quality of life of dogs as well as their owners (7–10). A recent study of 92 cases of canine pruritic flea infestation dermatitis showed that dog and owner quality of life improved significantly after a treatment course for this relatively common condition (10), highlighting a need to decrease this burden for dogs, dog owners, and veterinarians.

Recent advances in digital technology and their utility in animal health is a topic of growing interest in veterinary medicine (11). The use of wearable devices, particularly biosensors, is becoming established in animal health monitoring, providing useful health data for veterinarians (12–15). Several wearable sensors coupled with computerized analytics have been developed in an attempt to objectively measure pruritic behaviors in dogs (16–20). Studies with wearable sensors used various measures to assess pruritic activity, including measurements of piezo-electric voltage generated over certain blocks of time (17–19) and multidimensional high frequency data sampling combined with computer algorithms derived by a machine learning process (16, 20, 21). The piezo-electric voltage sensor lacks specificity (17–19) and the multidimensional sampling and algorithm technology tool is only able to detect scratching, head shaking, and sleep quality. Pruritus also includes behaviors such as paw licking and chewing (16, 20) which are not detected by wearables. Wearable activity monitor systems that can accurately and objectively detect changes in pruritic behaviors in dogs and send alerts of such changes to dog owners may offer an opportunity to improve dog health.

The Pet Insight Project (PIP) at Kinship, Inc. is a team of data scientists, technologists, and veterinarians, using artificial intelligence to link changes in behaviors with changes in pet health (22, 23). PIP was launched in 2018 to conduct one of the largest digital veterinary health studies designed to collect and interpret health information from hundreds of thousands of dogs with the aim to improve animal health by utilizing Whistle^®^ commercial dog activity monitors (22, 23). One of the canine activity monitors utilized in PIP is the Whistle FIT^®^ accelerometer (Mars Petcare, McLean, VA, United States). Over 100,000 Whistle FIT^®^ activity monitors have been distributed to dogs in PIP, with datasets containing over 24 million days in dogs’ lives that have enabled insights to support wellness of pets and improve veterinary care (23, 24). Data from PIP participants is used to train deep learning algorithms, creating FilterNet, a deep learning algorithm that has shown accuracy in activity recognition in real world use (24). Data from the Whistle activity monitors is processed by FilterNet and then transmitted to an application (app) on the owner’s tablet or smartphone (22). The app displays the processed dog activity data to the owner including specific pruritic activities of scratching and licking. This technology may offer a cost-effective strategy to increase the efficacy of veterinary care by overcoming the many significant barriers to understanding which dogs have pruritus, whether it is normal or abnormal, and whether veterinary intervention is necessary. Given that the Whistle FIT® monitor and smartphone/tablet app can detect changes in pruritic related activity in dogs such as scratching and self-licking (23), it is of interest to further evaluate how this system performs in real-world settings.

The current study was conducted as part of PIP which has provided an opportunity to perform detailed studies on how Whistle^®^ commercial pet activity monitors may affect dog owner behavior and pet health (22). Data from the Whistle FIT^®^ activity monitors/FilterNet algorithm system combined with owner-provided surveys and exams documented in the electronic health records (EHRs) at Banfield Pet Hospital clinics, provide opportunities to develop and validate proactive health tools, and to study how these health tools perform in real-life naturalistic settings. The capability to relay unbiased, objective dog behavior data to the owner via an alert may help to mitigate owner inexperience, lack of knowledge, and limited observational capacity.

Wearable activity monitor systems that can objectively detect increases and decreases in pruritic behaviors in dogs, and send alerts indicating such changes to dog owners, have been developed (16, 17, 19–23). The objectives of this retrospective, observational study were to investigate the impact of digital alerts for increased pruritic behaviors of scratching and licking received by dog owners in a natural real-life context, on (1) the propensity of dog owners to initiate a veterinary clinic visit, and (2) if such a clinic visit resulted in institution of therapy to manage pruritus. A preliminary report of data from this study has been published in abstract form (25).

## 2. Materials and methods

### 2.1. Study design

This was a retrospective, observational study based on a review of EHRs. Veterinary clinic visit behavior, diagnostic testing, and resulting prescriptions were derived from EHRs and compared with scratching and licking alerts sent to pet owners from a dog activity monitor system. The Strengthening the Reporting of Observational Studies in Epidemiology (STROBE) veterinary statement was used as a guide in creating this report (26).

### 2.2. Activity monitor and smartphone/tablet app

The Whistle FIT^®^ activity monitor can detect periods of activity in dogs (15). The Whistle FIT^®^ device is lightweight (14 g), compact (4 cm x 3.8 cm x 1 cm), water-resistant, battery-operated, and can attach to collars up to 2.54 cm wide (23). A deep learning computer algorithm (FilterNet) has been developed to analyze accelerometer data from the Whistle FIT® and validated to detect licking, scratching, sleeping, eating, and drinking, as well as fitness and movement (23, 24). At the time of this study, the system required Bluetooth, home WiFi (dual-band or 2.4 GHz), and a smartphone or tablet running iOS 10.0 or later or Android 5.0 or later.

The FilterNet algorithm in this study detected the characteristic accelerometer signal and identified it as a behavior like scratching or self-licking. Output data from the algorithm was analyzed by the Whistle system. A mobile app displayed the duration of time of scratching (seconds/day) and licking (minutes/day) as a rolling average of the previous 7 days of activity. Levels were quantified as infrequent (scratching, 0–52 s/day; licking, 0–7 min/day), occasional (53–119 s/day; licking, 7–19 min/day), elevated (scratching 120–299 s/day; licking, 19–43 min/day), and severe (scratching >300 s/day; licking, > 43 min/day). The scratching levels were previously validated with Pruritus Visual Analogue Scale (27). Survey validation with dog owners supported the licking categories that were developed; this internal validation has not been published at the time of writing. These categories were created by data collected during the Pet Insight Project. We evaluated population level scratching and licking metrics for dogs with healthy vs. unhealthy skin as indicated in an electronic health record. The control populations were also limited to only healthy pets to not add a confounding comorbidity such as osteoarthritis, which could increase licking levels in response to pain.

The Whistle system sent a digital message (pruritus alert) to the dog owners in the form of a push notification or e-mail when their dog’s scratching and/or licking activity had increased by one category level or more (e.g., from infrequent to occasional) based on the rolling average of the previous 7 days. The Whistle system required activity data present for a minimum of 3.5 days in the previous 7 days in order for the system to generate an alert for a dog. The system also requires a baseline period of 7 days of usage when a collar is first worn to establish starting scratching and licking levels. The earliest an alert could be sent is on day 9 of consecutive collar usage when first using the device.

### 2.3. Setting

**Figure 1:**
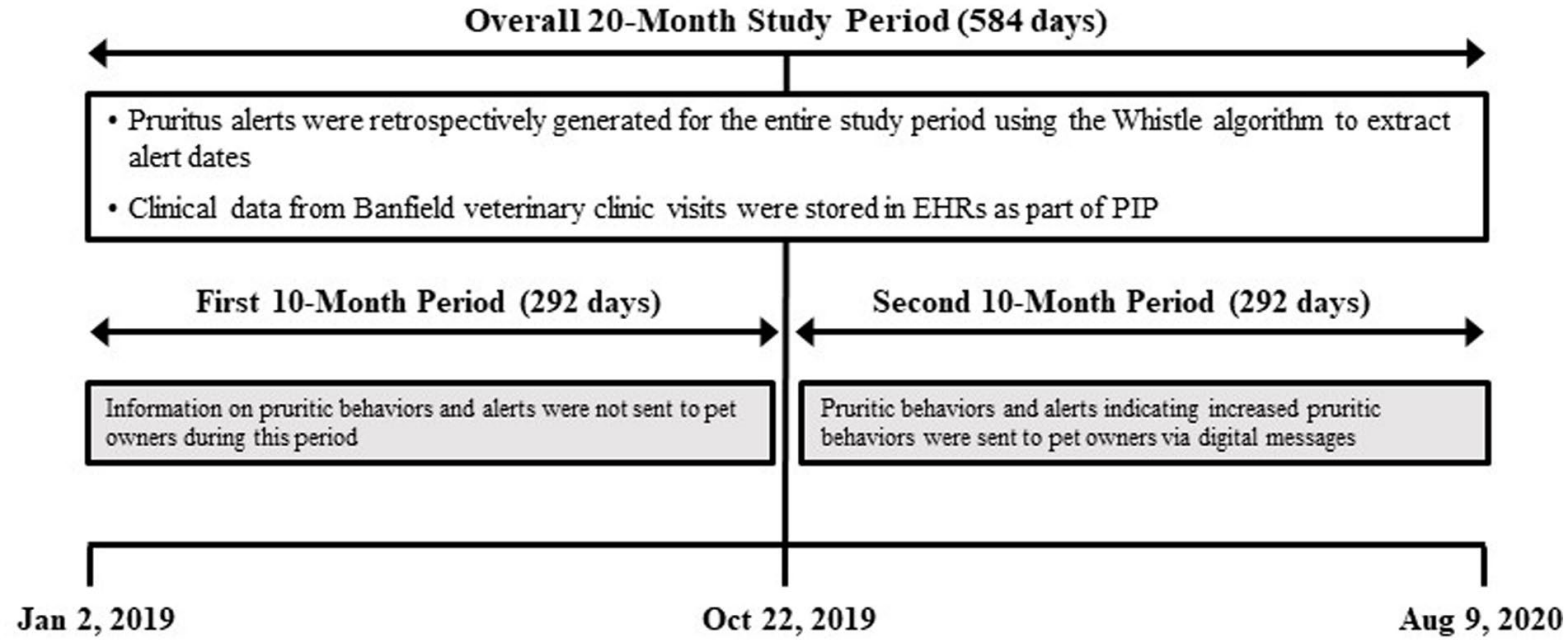
Schematic diagram of the study design. The overall study sampling period of 20 months (584 days) was divided into consecutive 10-month (292-day) periods. EHRs, electronic health records; PIP, Pet Insight Project.

All dogs in this study wore the Whistle FIT^®^ monitor as part of PIP. Monitor data were recorded and stored in the Whistle digital database maintained by PIP. Electronic health records from 1,042 Banfield veterinary clinics located in the United States and Puerto Rico were used to identify dogs included in this study. The Whistle digital database and Banfield EHRs were two separate and distinct data sources. Clinical data were obtained from Banfield EHRs via a Mars centralized database that is connected to the Banfield system. Dogs that had Whistle FIT^®^ monitor activity recorded during an approximate 20-month (584 days) study period, from January 2, 2019, to August 10, 2020, and Banfield EHRs from that same period, were screened for inclusion. The 20-month study period was divided into two 10-month (292 days) intervals, with a midpoint of October 2019 (Figure 1). This dataset did not include telemedicine consultations.

For the study period, the investigators applied the same algorithms used by the Whistle system to calculate the levels of pruritic behaviors (scratching and licking) and times when ‘alerts’ of increased activity would have been generated. Of important note, the dogs were wearing the same devices capturing raw data and the same methods of calculating and predicting scratching, licking, and alerts were used in the ‘pre’ and ‘post’ time periods. At the midpoint of the study period, October 2019, Pet Insight launched the first health feature in Whistle app. Dog owners could then see objective data about how much their dog was scratching or licking themselves that was not previously available in the first 10-month study period. Along with the visuals in the app, push notifications and emails of when dogs changed categories of scratching or licking were sent to dog owners (Supplementary Table S1). Logging of the alerts sent was not included in the Whistle system.

### 2.4. Participants

The source data were reviewed retrospectively to identify dogs with demographic data, veterinary clinic visits, and monitor activity during the 20-month study period. Eligible dogs had scratch and lick monitor activity recorded at some point during both the first and second 10-month intervals. The same dogs were included in both time periods however they may have only had a visit in one of these time periods.

### 2.5. Data collection

Each eligible dog was assigned a unique study number. Data evaluated included level of scratching and licking, clinic visit date, clinic visit outcome, demographics, and medical treatments prescribed (Table 1). Pruritus alerts were not stored by the Whistle system and instead were retrospectively generated for the entire study period using the Whistle algorithm to extract alert dates. The algorithm was applied to the activity monitor derived scratching and licking data collected during the study period.

**Table 1:** Data retrospectively collected and evaluated in this study.

Data categories	Variables
Pruritus behavior^a^	Level of scratching, level of licking (previous 7-day rolling average)
Pruritus alerts^a^	Increased scratching, increased licking, date sent
Clinic visit	Date of visit, outcome
Demographics	Age, breed, sex
Systemic Antipruritic treatment^b^	Treatment courses prescribed per clinic visit^c^
Antibiotic treatment (topical and/or systemic)^d^	Treatment courses prescribed per clinic visit^c^
Topical otic treatment^e^	Treatment courses prescribed per clinic visit^c^
Topical skin treatment^f^	Treatment courses prescribed per clinic visit^c^
Nutritionals^g^	Treatment courses prescribed per clinic visit^c^
Total treatments^h^	Treatment courses prescribed per clinic visit^c^

a Pruritus alerts were retrospectively generated for the entire study period using the Whistle algorithm to extract alert dates.

b Lokivetmab, chlorpheniramine, cyclosporine, dexamethasone, diphenhydramine, hydroxyzine, oclacitinib, prednisone, trimeprazine/prednisone.

c Each type of drug was regarded as one treatment course regardless of duration of treatment.

d Amoxicillin, amoxicillin/clavulanate, bacitracin/neomycin/polymyxin, cefadroxil, cefazolin, cefovecin, cefpodoxime, clindamycin, doxycycline, enrofloxacin, enrofloxacin/silver sulfadiazine, gentamicin/betamethasone, gentamicin/clotrimazole/mometasone, marbofloxacin, nystatin/neomycin/thiostrepton/triamcinolone acetonide, sulfadimethoxine/ormetoprim, sulfamethoxazole/trimethoprim.

e Acetic acid/boric acid, acetic acid/hydocortisone, ear cleaner non-medicated, enrofloxacin/silver sulfadiazine, florfenicol/terbinafine/betamethasone acetate, florfenicol/terbinafine/mometasone furoate, fluocinolone/dimethyl sulfoxide, gentamicin/betamethasone, gentamicin/clotrimazole/mometasone, ketoconazole/chlorhexidine/tris-EDTA, tromethamine/disodium EDTA dihydrate.

f Acetic acid/chlorhexidine/ketoconazole, benzoyl peroxide, benzoyl peroxide/sulfur/salicylic acid, chlorhexidine /climbazole, chlorhexidine/tromethamine/disodium EDTA dihydrate, conditioner non-medicated, hydrocortisone/aluminum acetate, nystatin/neomycin/thiostrepton/triamcinolone acetonide, sulfur/salicylic acid.

g Dermatology diet and fish oil/omega fatty acids/vitamin E.

h Includes all treatments in all categories listed in this table.

### 2.6. Variables evaluated

In addition to analyzing the total dataset, dogs were organized into four dermatitis subgroups based on the number of veterinary clinic visits with a dermatitis outcome during the first 10 months of the study. A veterinary clinic visit was determined to have a dermatitis outcome based on a ‘condition’ that matched a specific list of dermatitis diagnosis codes related to primary or secondary pruritus. Banfield has diagnostic codes that allow capture of a large number of conditions in a structured data set. All primary pruritic conditions, for example atopy or flea allergies, were selected for inclusion along with conditions such as pyoderma which may have been a secondary complication to a damaged skin barrier of any etiology. The list of structured diagnostic codes is not meant to be exhaustive, however it allows most conditions a practitioner will encounter to be captured as structured data (Supplementary Table S2). The dermatitis subgroups were defined as follows:

- Group 0: zero veterinary clinic visits with a dermatitis outcome.

- Group 1: one veterinary clinic visit with a dermatitis outcome.

- Group 2: 2 to 5 veterinary clinic visits with a dermatitis outcome.

- Group 3: 6 or more veterinary clinic visits with a dermatitis outcome.

The primary endpoint variable was the occurrence of a pruritic behavior ‘alert visit’, defined as the first veterinary clinic visit that occurred within 4 weeks after a pruritus alert. Veterinarians were not given any protocols to follow, they diagnosed and prescribed based on their evaluation of the history provided by the dog owner and the physical examination of the dog. Veterinary clinic visits that occurred prior to a pruritus alert, after an alert visit but still within the four-week window, or more than 4 weeks after a pruritus alert, were reported as veterinary clinic visits but not flagged as alert visits. Multiple visits that followed multiple alerts were treated as a unique alert with a resulting visit if a visit was within a 1-month period of the alert. The secondary endpoint variable was medications prescribed.

### 2.7. Statistical analysis

Descriptive statistics were used to summarize the datasets in this study. Odds ratios and 95% confidence intervals were calculated using the MedCalc Software Ltd. (28), according to Altman (29). Statistical significance of odds ratios was calculated according to Sheskin (30). All tests were two-tailed with an α value ≤0.05 indicating statistical significance.

## 3. Results

### 3.1. First and second 10-month study period datasets

Over 100,000 dogs were provided with Whistle FITs for PIP (22). EHRs, demographic data and clinic visits were available for 88,608 dogs. Of these, 24,174 dogs had demographic data and dermatologic-specific clinic visits during the study period. Of these, 10,951/24,174 (45%) dogs had data for demographics, veterinary clinic visits, and monitor activity (scratching or licking). Of these, 8,631/10,951 (79%) dogs had monitor activity recorded at some point during both the first and second 10-month intervals of the 20-month study period. Of these, 7,191/8631 (83%) dogs had a veterinary clinic visit and at least one pruritus alert during the first 10-month period and 6684/8631 (77%) dogs had a veterinary clinic visit and at least one pruritus alert during the second 10-month period (Table 2). A total of 6183/8631 (72%) dogs were included in both study periods.

**Table 2:** Summary of eligible dogs evaluated in each period of the study.

Stage of study	Number of dogs / Dermatitis subgroup assignment^a^
Dogs distributed a Whistle FIT® monitor were assessed for eligibility	*n* ~ 100,000
Dogs with EHRs, demographic data and veterinary clinic visits	*n* = 88,608
Dogs with demographic data and dermatologic-specific veterinary clinic visits during the 20-month study period (Jan 2, 2019 to Aug 9, 2020)	*n* = 24,174
Dogs with Whistle FIT® monitor activity (scratching or licking) during the 20-month study period (Jan 2, 2019 to Aug 9, 2020)	*n* = 10,951
Dogs with Whistle FIT® monitor activity (scratching or licking) during both the first and second 10-month intervals of the 20-month study period	*n* = 8,631
Total dogs included in the first 10-month interval (Whistle FIT® monitor activity plus a veterinary clinic visit)	*n* = 7,191 Group 0, *n* = 2,845 (39.6%) Group 1, *n* = 2,495 (34.7%) Group 2, *n* = 1,684 (23.4%) Group 3, *n* = 167 (2.3%)
Dogs that had Whistle FIT® monitor activity (scratching or licking) but did not have a veterinary clinic visit for any reason during the second 10-month interval	*n* = − 1,008
Dogs that had Whistle FIT® monitor activity (scratching or licking) but did not have a veterinary clinic visit for any reason during the first 10-month interval^b^	*n* = + 501 Group 0, *n* = 501
Total dogs included in the second 10-month interval (Whistle FIT® monitor activity plus a veterinary clinic visit)	*n* = 6,684 Group 0, *n* = 3,062 (45.8%) Group 1, *n* = 2025 (30.3%) Group 2, *n* = 1,440 (21.5%) Group 3, *n* = 157 (2.3%)

EHRs, Electronic health records.

a Dermatitis subgroup assignment based on the number of veterinary clinic visits with a dermatitis outcome during the first 10 months of the study: Group 0, 0 visits with dermatitis outcome; Group 1, 1 visit with dermatitis outcome; Group 2, 2–5 visits with dermatitis outcome; Group 3, ≥ 6 visits with dermatitis outcome.

b These dogs were assigned to Group 0 for all subgroup analyses.

The signalment of dogs was similar in the first and second 10-month study periods (Supplementary Table S3). Mean (SD) ages of dogs were 4.3 (3.4) years and 4.4 (3.4) years in the first and second 10-month study periods, respectively. Approximately half of the dogs in both 10-month periods were male, over 90% of the males were neutered; and over 95% of the females were spayed. Toy, sporting, herding, and working groups each comprised greater than 10% of dogs in both study periods.

During the first 10-month period (Supplementary Table S4), a total of 113,530 alerts of increased scratching and/or licking were generated retrospectively by the investigators, with an average of 15.8 alerts per dog (median = 9.0, range = 60, max = 61). During the second 10-month study period, a total of 93,217 alerts were generated by the Whistle system, with an average of 13.9 alerts per dog (median = 11.0, range = 45, max = 46).

### 3.2. Alert visits

The primary endpoint (occurrence of a pruritic behavior alert visit) was evaluated relative to the total number of pruritus alerts and by the total number of veterinary clinic visits. The relationships between pruritus alerts associated with alert visits and the total number of alerts generated are summarized in Tables 3, 4. The frequency of alerts associated with an alert visit ranged from 2% (status = severe scratching/infrequent licking) to 8.8% (status = severe scratching/severe licking) in the first 10 months of the study, and from 5.5% (status = occasional scratching/infrequent licking) to 16% (status = severe scratching/severe licking) in the second 10 months (Table 3). In the first 10-month period, 4.74% (5,382/113,530) of all alerts for increased scratching or licking were associated with an alert visit compared with 7.49% (6,980/93,217) in the second 10-month period (Table 4). The odds of an alert visit were statistically significantly more likely (odds ratio, 1.6264; 95% CI, 1.57–1.69; *p* < 0.0001) in the second 10-month study period compared with the first 10-month study period, for all levels of scratching (Table 5) and licking (Table 6).

**Table 3:** Number of pruritus alerts associated with a veterinary clinic visit within four weeks after the date of the alert.^a^

	First 10-month study period (*n* = 7,191 dogs)				Second 10-month study period^b^ (*n* = 6,684 dogs)			
	Licking/infrequent	Licking/occasional	Licking/elevated	Licking/severe	Licking/infrequent	Licking/occasional	Licking/elevated	Licking/severe
Scratching/infrequent	N/A	358/9934 (3.6%)	294/7974 (3.7%)	21/431 (4.9%)	N/A	1119/14,595 (7.7%)	877/11,244 (7.8%)	297/2868 (10%)
Scratching/occasional	128/4351(2.9%)	1429/32,002 (4.5%)	956/19,772 (4.8%)	46/794 (5.8%)	390/7050 (5.5%)	919/15,848 (5.8%)	715/10,541 (6.8%)	257/2837 (9.1%)
Scratching/elevated	46/1574 (2.9%)	855/16,575 (5.2%)	861/13,795 (6.2%)	57/913 (6.2%)	210/3257 (6.4%)	559/7903 (7.1%)	540/6715 (8%)	234/2480 (9.4%)
Scratching/severe	4/197 (2%)	93/1992 (4.7%)	192/2746 (7%)	42/480 (8.8%)	65/834 (7.8%)	193/2382 (8.1%)	314/2884 (11%)	291/1779 (16%)

Data are number of alerts associated with a veterinary clinic visit within four weeks/total number (%) of alerts generated within the 10-month period. Each cell represents the status of scratching and licking when the alert was generated. N/A, not applicable.

a Pruritus alerts were retrospectively generated for the entire study period using the Whistle algorithm to extract alert dates. An alert was generated whenever the scratching or licking activity increased by one or more category levels from the previous day (e.g., from infrequent to occasional).

b In the second 10-month period, pruritic behaviors and alerts indicating increased pruritic behaviors were sent to pet owners via digital messages.

**Table 4:** Pruritus alert data from Table 3 shown by the level of scratching or licking when the alert was generated.^a^

Alert category	First 10-month study period (*n* = 7,191 dogs)	Second 10-month study period^d^ (*n* = 6,684 dogs)
Scratching - infrequent^b^	673/18,339 (3.67%)	2293/28,707 (7.99%)
Scratching - occasional	2559/56,919 (4.50%)	2281/36,276 (6.29%)
Scratching - elevated	1819/32,857 (5.54%)	1543/20,355 (7.58%)
Scratching - severe	331/5415 (6.11%)	863/7879 (10.95%)
Total: all scratching levels combined	5382/113,530 (4.74%)	6980/93,217 (7.49%)
Licking - infrequent^c^	178/6122 (2.9%)	665/11,141 (6.0%)
Licking - occasional	2735/60,503 (4.5%)	2790/40,728 (6.9%)
Licking - elevated	2303/44,287 (2.9%)	2446/31,384 (7.8%)
Licking - severe	166/2618 (6.3%)	1079/9964 (10.8%)
Total: all licking levels combined	5382/113,530 (4.74%)	6980/93,217 (7.49%)

Data are number of alerts associated with a veterinary clinic visit within four weeks/total number (%) of alerts generated within the 10-month period.

a Pruritus alerts were retrospectively generated for the entire study period using the Whistle algorithm to extract alert dates. An alert was generated whenever the scratching or licking activity increased by one or more category levels from the previous day (e.g., from infrequent to occasional).

b Alerts categorized in the infrequent scratch category in this table are the result of an increase in lick behaviors, as alerts can be sent for increases in scratch and/or lick.

c Alerts categorized in the infrequent lick category in this table are the result of an increase in scratch behaviors, as alerts can be sent for increases in lick and/or scratch.

d In the second 10-month period, pruritic behaviors and alerts indicating increased pruritic behaviors were sent to pet owners via digital messages.

**Table 5:** Two by two tables and odds ratios for pruritus alerts by level of scratching at time of alert.^a^

Scratching level	Impact of alert^c^	Second 10-month study period^d^ (exposure)	First 10-month study period (control)	Odds ratio	95% CI	*p* value
Infrequent^b^	Associated with a 4-week veterinary clinic visit	2,293	673	2.28	2.09–2.49	<0.0001
	Not associated with a 4-week veterinary clinic visit	26,414	17,666			
Occasional	Associated with a 4-week veterinary clinic visit	2,281	2,559	1.43	1.35–1.51	< 0.0001
	Not associated with a 4-week veterinary clinic visit	33,995	54,360			
Elevated	Associated with a 4-week veterinary clinic visit	1,543	1819	1.40	1.30–1.50	< 0.0001
	Not associated with a 4-week veterinary clinic visit	18,812	31,038			
Severe	Associated with a 4-week veterinary clinic visit	863	331	1.89	1.66–2.16	< 0.0001
	Not associated with a 4-week veterinary clinic visit	7,016	5,084			
All Categories Combined (Total)	Associated with a 4-week veterinary clinic visit	6,980	5,382	1.6264	1.57–1.69	< 0.0001
	Not associated with a 4-week veterinary clinic visit	86,237	108,148			

Data are number of alerts; data grouped by scratching level paired with any level of licking.

a Pruritus alerts were retrospectively generated for the entire study period using the Whistle algorithm to extract alert dates. An alert was generated whenever the scratching or licking activity increased by one or more category levels from the previous day (e.g., from infrequent to occasional). Each row represents the level of scratching when the alert was generated.

b Alerts categorized in the infrequent scratch category in this table are the result of an increase in lick behaviors, as alerts can be sent for increases in scratch and/or lick.

c The alert either was or wasn’t associated with a veterinary clinic visit within 4 weeks after the date of the alert.

d In the second 10-month period, pruritic behaviors and alerts indicating increased pruritic behaviors were sent to pet owners via digital messages.

**Table 6:** Two by two tables and odds ratios for pruritus alerts by level of licking at time of alert.^a^

Licking level	Impact of alert^c^	Second 10-month study period^d^ (exposure)	First 10-month study period (control)	Odds ratio	95% CI	*p* value
Infrequent^b^	Associated with a 4-week veterinary clinic visit	665	178	2.12	1.79–2.51	< 0.0001
	Not associated with a 4-week veterinary clinic visit	10,476	5,944			
Occasional	Associated with a 4-week veterinary clinic visit	2,790	2,735	1.55	1.47–1.64	< 0.0001
	Not associated with a 4-week veterinary clinic visit	37,938	57,768			
Elevated	Associated with a 4-week veterinary clinic visit	2,446	2,303	1.54	1.45–1.63	< 0.0001
	Not Associated with a 4-Week Veterinary Clinic Visit	28,938	41,984			
Severe	Associated with a 4-week veterinary clinic visit	1,079	166	1.79	1.51–2.13	< 0.0001
	Not associated with a 4-week veterinary clinic visit	8,885	2,452			
All Categories Combined (Total)	Associated with a 4-week veterinary clinic visit	6,980	5,382	1.6264	1.57–1.69	< 0.0001
	Not associated with a 4-week veterinary clinic visit	86,237	108,148			

Data are number of alerts; data grouped by licking level paired with any level of scratching.

a Pruritus alerts were retrospectively generated for the entire study period using the Whistle algorithm to extract alert dates. An alert was generated whenever the scratching or licking activity increased by one or more category levels from the previous day (e.g., from infrequent to occasional). Each row represents the level of licking when the alert was generated.

b Alerts categorized in the infrequent lick category in this table are the result of an increase in scratch behaviors, as alerts can be sent for increases in lick and/or scratch.

c The alert either was or wasn’t associated with a veterinary clinic visit within 4 weeks after the date of the alert.

d In the second 10-month period, pruritic behaviors and alerts indicating increased pruritic behaviors were sent to pet owners via digital messages.

**Figure 2:**
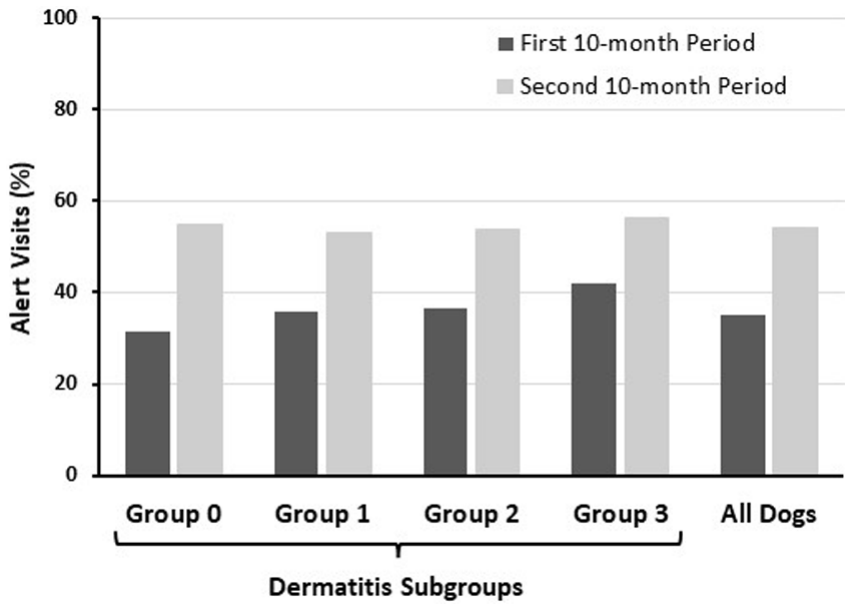
Alert visits as a percentage of total visits during each study period (data from Table 7). Alert visits were defined as the first clinic visit that occurred within 4 weeks after the date of a pruritus alert. The frequencies of alert visits were higher in the second 10 months compared with the first 10 months in each dermatitis subgroup and in the overall group data.

Veterinary clinic visit data are summarized in Table 7 and Figure 2. For all dogs, 35.2% (5,382/15,278) of veterinary clinic visits were alert visits (occurred within 4 weeks after a pruritus alert) in the first 10-month period compared with 54.1% (6,980/12,900) in the second 10-month period. A majority of alert visits occurred within 1 week of a pruritus alert in each dermatitis subgroup of dogs, in both the first and second 10-month periods (Table 7). The frequency of alert visits was higher in the second 10-month period than in the first 10-month period for each dermatitis subgroup of dogs (Table 7; Figure 2). Dogs in Group 0 had the highest number of alert visits in the second 10-month period (Table 7).

**Table 7:** Summary of veterinary clinic visits by dermatitis subgroup and study period.

Dog dermatitis subgroup^a^	Number (%) of veterinary clinic visits by time interval (time after pruritus alert)					Veterinary visits outside of the 4-week window	Total visits at any time during the study period
	1 week	2 weeks	3 weeks	4 weeks	Sum of weeks 1-4^b^		
First 10-month study period							
Group 0 (*n* = 2,845 dogs)	971	258	77	33	1,339 (31.4%)	2,926 (68.6%)	4,265
Group 1 (*n* = 2,495 dogs)	1,179	300	103	52	1,634 (35.7%)	2,939 (64.3%)	4,573
Group 2 (*n* = 1,684 dogs)	1,380	362	134	58	1,934 (36.5%)	3,370 (63.5%)	5,304
Group 3 (*n* = 167 dogs)	358	87	14	16	475 (41.8%)	661 (58.2%)	1,136
Total (*n* = 7,191 dogs)	3,888	1,007	328	159	5,382 (35.2%)	9,896 (64.8%)	15,278
Second 10-month study period							
Group 0 (*n* = 3,062 dogs)	1,632	724	469	294	3,119 (54.7%)	2,582 (45.3)	5,701
Group 1 (*n* = 2025 dogs)	937	497	289	179	1902 (53.2%)	1,672 (46.8%)	3,574
Group 2 (*n* = 1,440 dogs)	855	400	225	151	1,631 (53.6%)	1,411 (46.4%)	3,042
Group 3 (*n* = 157 dogs)	202	69	36	21	328 (56.3%)	255 (43.7%)	583
Total (*n* = 6,684 dogs)	3,626	1,690	1,019	645	6,980 (54.1%)	5,920 (45.9%)	12,900

a Dermatitis subgroups were based on the number of veterinary clinic visits with a dermatitis outcome during the first 10 months of the study. Group 0: 0 visits with dermatitis outcome; Group 1: 1 visit with dermatitis outcome; Group 2: 2–5 visits with dermatitis outcome; Group 3: ≥ 6 visits with dermatitis outcome.

b 28-day period. The number of visits in this column are the primary endpoint variable, which was the occurrence of a pruritic behavior ‘alert visit’, defined as the first veterinary clinic visit that occurred within 4 weeks after a pruritus alert.

### 3.3. Dermatologic medication prescribing patterns

For the secondary endpoint evaluation of the medications prescribed during the first and second 10-month study periods, data included the following five therapeutic categories: antipruritics, antibiotics, otics, topicals, and nutritionals. Overall, more medications were prescribed in the first 10-month study period (*n* = 10,829 medications) compared to the second 10-month study period (*n* = 9,863 medications, Supplementary Table S5). However, the number of medications prescribed per dog in the first 10-month period (1.51) was similar to the second 10- month period (1.49). In the first 10-month study period, 38.8% (4,201/10,829) of medications were prescribed within 4 weeks after a pruritus alert. In the second 10-month study period 55.3% (5,457/9863) of medications were prescribed within 4 weeks after a pruritus alert. In the subgroup of dogs with zero veterinary clinic visits with a dermatitis outcome (Group 0), the number of medications prescribed in the first 10 months (*n* = 894 medications) was lower than the second 10 months (*n* = 3,921 medications). In contrast, in all other subgroups of dogs (Groups 1–3), the number of medications prescribed in the first 10 months was higher than the number of medications prescribed in the second 10 months.

Medications prescribed to dogs in Group 0 are summarized in Supplementary Table S6. In the first 10 months, the percentage of medications prescribed within 4 weeks after a pruritus alert, by category, ranged from 31.7% (topicals) to 33.3% (antipruritics and otics), compared with a range of 46.6% (nutritionals) to 59.4% (antipruritics) in the second 10 months. The total number of medications prescribed in each category was higher in the second 10-month period compared to the first 10-month period. For each medication category, the increase in number of medications prescribed, from the first to second 10-month periods, were as follows: antipruritics, 113 to 1,067; antibiotics, 91 to 640; otics, 113 to 470; topicals, 13 to 263; and nutritionals, 10 to 41.

## 4. Discussion

This was a retrospective study of the relationship between alerts of increased pruritic activity in dogs, detected by a collar-mounted activity monitor and received by dog owners, and the action of the owners to take their dog to a veterinary clinic visit, in a natural, real-world setting. Dogs in this study wore a Whistle FIT^®^ collar monitor as part of the ongoing PIP. Activity data and veterinary EHR data recorded in over 7,000 dogs were evaluated retrospectively for a 20-month period. For the first 10 months of this 20-month period, the Whistle system component for detecting the pruritic behaviors of scratching and self-licking was not yet in operation. At the midpoint of the 20-month period, the pruritus detection component was launched. Dog owners could then view reports on their pet’s pruritic activity in an app and receive digital alerts of increased levels of scratching and/or licking via a smartphone/tablet app and e-mail. Records of the pruritus alerts were not stored by the Whistle system. For the purposes of this study, the investigators applied the same algorithms used by the Whistle system to retrospectively calculate pruritic behavior categories and times when alerts of increased pruritic behaviors would have been generated by the Whistle system for the entire study period. Data from the first 10-month period served as a control and was compared to the second 10-month period.

The total number of pruritic alerts was 113,530 in the first 10 months and 93,217 in the second 10 months. The number of veterinary clinic visits within 4 weeks after an alert was higher in the second 10-month period (7.49%) compared to the first 10-month period (4.74%) for the overall dataset, and for each level of scratching and licking. The odds of an alert visit were statistically significantly more likely (odds ratio, 1.6264; 95% CI, 1.57–1.69; *p* < 0.0001) in the second 10-month interval when the dog owner was sent a digital alert compared with the first 10-month interval when the dog owner was not sent a digital alert. Similar trends were seen in the visit data, and in the secondary endpoint of dermatitis medications prescribed. The overall number of veterinary clinic visits was lower in the second 10 months (*n* = 12,900) compared to the first 10 months (*n* = 15,278), possibly because of the SARS-CoV-2 pandemic. However, the number of clinic visits within 4 weeks after a pruritus alert was higher in the second 10 months (6,980/12,900, 54.1%) than in the first 10 months (5,382/15,278, 35.2%) for the overall dataset, and for each dermatitis subgroup of dogs. The total number of medications prescribed was lower in the second 10 months (*n* = 9,863) compared to the first 10 months (*n* = 10,829). In contrast, the percentage of medications prescribed within 4 weeks after a pruritus alert was higher in the second 10-month period (53.3%) compared to the first 10-month period (38.8%).

The primary aim of this study was to better understand how dog owners respond to pruritic behavior alerts received from a collar-mounted accelerometer paired with a smartphone/tablet app in a real-world setting. Results suggest that dog owners may be more inclined to take action when provided with quantitative information about changes in pruritic behavior in their dogs, particularly for dogs that did not have a clinical history of pruritus (the cohort of dogs that were not diagnosed with a dermatologic condition in the first 10-month period of the study; Group 0). The Whistle system may be associated with changes in dog owner behavior as evidenced by a markedly higher frequency of veterinary clinic visits taking place within 4 weeks after an alert was sent to the dog owner from the system in the second 10 months of the study period. For the primary and secondary endpoints, a four-week (28-day) window after a pruritus alert was selected for analyses based on previous experience with the Whistle system, which has shown that veterinary clinic visits decrease rapidly over a 4-week period after an alert. This trend was also seen in the current study. It has also been shown that dog owners respond relatively quickly to signs of pruritic activity in their dogs. A recent study of 92 dogs with flea infestation dermatitis showed that the average (SD) time between clinical signs of pruritus in dogs and a veterinary visit was 5.4 (3.9) days (range, 1–30 days), and a treatment course for this common type of dermatitis resulted in a significant improvement in dog and owner quality of life (*p* < 0.001), assessed with an 8-item canine dermatitis quality of life questionnaire (10). Because canine pruritus negatively impacts the quality of life of dogs and their owners (7–10), it would not be practical for the owners to wait longer than 4 weeks to seek veterinary care once the pruritic activity is recognized.

Results for the secondary endpoint of dermatitis medications showed that the total number of pruritus-related medications prescribed during the second 10-month period (*n* = 9,863) was lower than during the first 10-month period (*n* = 10,829). The number of medications was also lower in the second 10 months compared to the first 10 months for all dermatitis subgroups of dogs except for Group 0, the subgroup with no veterinary clinic visits with a dermatitis outcome. In Group 0, 894 medications were prescribed to 2,845 dogs in the first 10 months compared to 3,921 medications prescribed to 3,062 dogs in the second 10 months. These results suggest that the Whistle system alerts may have increased dog owner and veterinarian awareness of pruritus in this subgroup, which may in turn have led to more proactive treatment of pruritus in these dogs. There was a trend in Groups 1–3 for fewer pruritus-related medications prescribed in the second 10 months. Owners of dogs in Groups 1–3 may have responded to Whistle system alerts in the second period by altering their vigilance and compliance with previously prescribed medications, in place of initiating a veterinary visit.

This study supports the concept that data generated from wearable activity monitors and analyzed by deep learning computer algorithms can serve as a proactive health tool for dog owners and veterinarians and may impact dog health. Another study that compared scratching severities recorded by the Whistle FIT^®^ monitor with severity scores determined by a pruritus visual analogue scale recorded by 358 dog owners reported statistically significant associations between the two scoring evaluations (27). The negative burden on pet owners that results from caring for a chronically ill companion animal has been described, with appearance of pain or discomfort as one of the signs/behaviors that correlates with owner burden (31). Two modifiable risk factors have been found to be associated with pet owner burden: reaction to companion animal clinical signs and owner sense of control (31). An accessible technology such as the Whistle system (24) combined with a dog’s clinical history, creates a new tool for owners to better detect clinical signs of pruritus, thereby potentially increasing their sense of control regarding their dog’s health and decreasing burden. An open question remains as to how activity tracker derived pruritic alerts impact caregiver burden for owners and veterinarians, to be addressed by future research efforts.

This retrospective review of client-owned dogs from PIP examined a relatively large number of dogs over an extended period (20 months) in which dogs were maintained in their home setting. Previous studies with wearable dog activity monitors (16–20) included relatively small sample sizes, ranging from six (20) to 361 (16) dogs and relatively short study periods, ranging from 7 days (17) to 8 weeks (18, 20). Data generated with the wearable activity monitors and computer software/algorithms utilized in prior studies lacked specificity (17–19) and did not provide information on pruritic activities other than scratching and head shaking (16, 20). The current study included over 7,000 dogs, with an activity monitor that has been developed in over 100,000 dogs and a deep learning algorithm that has been trained over a period of 2–3 years (23, 24). The demographics, geographic distribution, and large number of the dogs evaluated in the current study support the inference that the dogs studied here may be generalizable to the broader population of pet dogs in the United States.

Limitations of this study include the retrospective, observational study design. It is not possible to establish a cause-and-effect relationship retrospectively. The potential influence of dog breed on the results was not determined in this study and remains an open question for future analyses. Additionally, there is potential for selection bias in data collection in retrospective studies as well as variability in the dataset (26). The dog owners in the present study may have introduced a selection bias. Dog owners who enroll in the PIP and obtain activity trackers for their dogs may be more engaged with their pets, and may not be generalizable to all dog owners.

The study population included 7,191 dogs in the first 10-month dataset and 6,684 dogs in the second 10-month dataset. This drop off may have been due to the confounding impact of widespread implementation of SARS-CoV-2 pandemic-related restrictions in the first half of 2020. Reports in veterinary medicine suggest that restrictions associated with the SARS-CoV-2 pandemic have had a substantial impact on animal health (32, 33), owners’ perceptions (32), and the veterinary profession (34). Some owners postponed veterinary visits due to the required separation from their dog during consultations and only sought care for their pet in emergency cases (32). Telemedicine visits may have been accessed by some owners; these visits were not tracked in our data. Financial constraints during the SARS-CoV-2 pandemic also led to delaying veterinary visits for some dog owners (32). Postponement of veterinary visits can lead to a missed diagnosis and lack of treatment for a health problem (32, 33). Dog owners may have also been more closely aware of their pets’ behaviors during the SARS-CoV-2 isolation, which could have impacted the results of this study. We recognize that owner behaviors are an important and complex topic that was not a focus of the current study. Future studies that specifically address owner behaviors, and how dog owners respond to receiving digital messages regarding pet health, are needed to more fully determine the utility of the Whistle system in managing pruritus in dogs.

The results of this study suggest that transmitting alerts to a dog owner’s smartphone app or computer by the Whistle system may help dog owners to recognize pruritic behaviors in their dogs, prompt changes in dog owner behavior, and increase the likelihood of a veterinary visit in response to receiving the alert. Pet owners in this study appeared to be highly motivated to manage their dogs’ pruritus and sought veterinary treatment when alerted to increases in their dogs’ pruritic behaviors, particularly in dogs without a history of pruritus. The Whistle system may help to decrease the burden of pruritus for dogs, their owners, and veterinarians.

## Data availability statement

The raw data supporting the conclusions of this article will be made available by the authors, without undue reservation.

## Ethics statement

The animal study was reviewed and approved by the WALTHAM Animal Welfare and Ethical Review Body (Project Portfolio Management number 58565, June 2017) and conducted under the authority of the Animals (Scientific Procedures) Act 1986. Written informed consent was obtained from the owners for the participation of their animals in this study.

## Author contributions

AC, CK, and AW contributed to conception and design of the study. CK, TR, and KW organized the database. TR and KW performed the statistical analysis. AC, CK, TR, KW, AW, and AH contributed to the interpretation of data for the work. All authors contributed to the article and approved the submitted version.

## Funding

This work was supported by Zoetis Inc. and Kinship Partners Inc.

## Conflict of interest

AC and CK are employees of At-Home Diagnostics, Mars Science and Diagnostics. TR and KW are employed by the University of Surrey. AW and AH are employed by Zoetis. This study received funding from Zoetis. The funder had the following involvement with the study: Support for University of Surrey analysis and manuscript preparation.

## Publisher’s note

All claims expressed in this article are solely those of the authors and do not necessarily represent those of their affiliated organizations, or those of the publisher, the editors and the reviewers. Any product that may be evaluated in this article, or claim that may be made by its manufacturer, is not guaranteed or endorsed by the publisher.
