# Supplementary material for: Response of pet owners to Whistle FIT® activity monitor digital alerts of increased pruritic activity in their dogs: a retrospective observational study
Source: Front Vet Sci. 2023 Aug 9;10:1123266. doi: 10.3389/fvets.2023.1123266 (PMC10445133; doi:10.3389/fvets.2023.1123266)
Supplement: Supplementary file 1 [file Data_Sheet_1.docx]

Supplementary Material

Response of pet owners to Whistle FIT® activity monitor digital alerts of increased pruritic activity in their dogs: A retrospective observational study

Aletha Carson*, Cassie Kresnye, Taranpreet Rai, Kevin Wells, Andrea Wright, Andrew Hillier

*** Correspondence:** Aletha Carson: [aletha.carson@kinship.co](mailto:aletha.carson@kinship.co)

# Supplementary Tables

**Supplementary Table 1.** Description of digital message notifications sent from the Whistle system to dog owners.

| **Context:**  The push notification and email alerts did not contain a call to action for the dog owner. The alert text stated the following: | |
| --- | --- |
| “(Dog name) has been (scratching/licking) more, (his/her) (scratching/licking) has increased to (scratch/lick category). Check out (his/her) health report to learn more.” | |
| This message included links to additional information to help owners more fully understand the context of the alert and what it might mean for their dog. | |
| If users looked at their health report in the app after receiving the prompt, they would see the text listed below, that included a recommendation to see a veterinarian in some cases. | |
| **Scratching** | **Licking** |
| **Infrequent**   - Less than 52 seconds of scratching per day with a 7-day average - Less scratching than 50% of other dogs - Infrequent scratching likely means your dog does not have an allergy or infection - What to do: Just keep tracking and doing what you are doing | **Infrequent**   - Fewer than 7 minutes of daily licking with a 7-day average - Less licking than 50% of other dogs - Infrequent licking likely means your dog has a lower risk of skin infections and allergies - What to do: Just keep tracking and doing what you’re doing |
| **Occasional**   - Between 53 and 119 seconds of scratching per day with a 7-day average - More scratching than the average dog. Occasional scratching could point to a skin issue or may just mean your dog likes a scratch more than most - What to do: Keep track of their scratching for any increases, which could be an early sign of a skin issue | **Occasional**   - Between 8-19 minutes of daily licking with a 7-day average - More licking than the average dog, but less than the top 85% of dogs with a higher rate of skin issues - Occasional licking could point to a skin issue or may just mean your dog likes to lick more than most - What to do: Keep tracking their licking for any changes or increases |
| **Elevated**   - Between 120 and 299 seconds of scratching per day with a 7-day average - Scratches more than 85% of dogs - Elevated scratching may be a symptom of skin issues like allergies and infections. It can also lead to skin damage - What to do: Consider booking a skin checkup. You can share scratching data with the vet, straight from the app | **Elevated**   - Between 20-43 minutes of daily licking with a 7-day average - More licking than 85% of other dogs - Elevated licking may be a symptom of skin issues like allergies and infections. It can also lead to skin damage. - What to do: Consider booking a skin checkup. You can share licking data with the vet straight from the app |
| **Severe**   - More than 300 seconds of daily scratching with a 7-day average - More scratching than 97% of other dogs. Severe scratching is almost always a sign of irritation. And it’s likely to cause more damage and discomfort - What to do: Book a skin checkup if you can. You can share scratching data with the vet, straight from the app | **Severe**   - More than 44 minutes of daily licking with a 7-day average - More licking than 97% of other dogs - Severe licking is almost always a sign of irritation. And it’s likely to cause more damage and discomfort - What to do: Book a skin checkup if you can. You can share licking data with the vet, straight from the app |

**Supplementary Table 2.** List of diagnostic codes/ailments (related to primary or secondary pruritus) recorded in the EHR and used to designate a veterinary clinic visit as having a dermatitis outcome.

| - Acariasis | - Dermatitis, Pyotraumatic | - Pruritus |
| --- | --- | --- |
| - Acne | - Dermatitis, Solar | - Pyoderma, Callus |
| - Alopecia, Endocrine | - Dermatitis, Tail Fold | - Pyoderma, Deep |
| - Atopy | - Dermatological Disease, Undetermined | - Pyoderma, Generalized |
| - Cellulitis, Conservative | - Dermatomycosis | - Pyoderma, Juvenile |
| - Cellulitis, Extensive | - Discoid Lupus Erythematosus | - Pyoderma, Nasal Fold |
| - Cheyletiella | - Ear Margin Dermatosis | - Pyoderma, Perivulvar |
| - Cutaneous Myiasis | - Fleas | - Pyoderma, Superficial |
| - Demodex | - Folliculitis | - Sarcoptic Mange |
| - Demodex, Generalized | - Food Allergy Dermatitis | - Sebaceous Adenitis |
| - Demodex, Localized | - Golden Retriever Ichthyosis | - Seborrhea |
| - Dermatitis | - Granuloma | - Seborrhea Oleosa |
| - Dermatitis, Atopic Allergic | - Granuloma, Acral Lick | - Seborrhea Sicca |
| - Dermatitis, Contact | - Impetigo | - Staph. Pyoderma |
| - Dermatitis, Flea Allergy | - Lichenification | - Tick Dermatitis |
| - Dermatitis, Food Allergy | - Malassezia | - Ticks |
| - Dermatitis, Idiopathic | - Paronychia | - Urticaria |
| - Dermatitis, Malassezia | - Pediculosis | - Vitamin A Responsive Dermatosis |
| - Dermatitis, Moist | - Pododermatitis/Interdigital Derm | - Zinc Responsive Dermatosis |

EHR, Electronic health record.

**Supplementary Table 3.** Signalment of the dogs.

| Signalment | | First 10-month Study Period  (n = 7191) | Second 10-month Study Period  (n = 6684) |
| --- | --- | --- | --- |
| Age, mean (SD) (years) | | 4.3 (3.4) | 4.4 (3.4) |
| Age, median (range) (years) | | 4 (0-18) | 4 (0-18) |
| Male, n (%) | | 3921 (54.5) | 3642 (54.5) |
|  | Neutered, n (%) | 3667 (93.5) | 3440 (94.5) |
|  | Intact, n (%) | 254 (6.5) | 202 (5.5) |
| Female, n (%) | | 3270 (45.5) | 3042 (45.5) |
|  | Spayed, n (%) | 3162 (96.7) | 2944 (96.8) |
|  | Intact, n (%) | 108 (3.3) | 98 (3.2) |
| Breeds, n (%) | | | |
|  | Toy group | 1329 (18.5) | 1243 (18.6) |
|  | Sporting group | 1311 (18.2) | 1238 (18.5) |
|  | Herding group | 916 (12.7) | 837 (12.5) |
|  | Working group | 795 (11.1) | 731 (10.9) |
|  | Terrier group | 709 (10.0) | 649 (9.7) |
|  | Non-sporting group | 604 (8.4) | 558 (8.3) |
|  | Hound group | 554 (7.7) | 537 (8.0) |
|  | Mixed breed | 464 (6.5) | 443 (6.6) |
|  | Pit bull | 342 (4.8) | 294 (4.4) |
|  | Foundation stock service | 140 (1.9) | 124 (1.9) |
|  | Not Recognised | 16 (0.2) | 16 (0.2) |
|  | Miscellaneous class | 11 (0.2) | 14 (0.2) |

**Supplementary Table 4.** Number of pruritic alerts indicating an increase in activity by one category level or more of scratching and/or licking. Each row represents the status of scratching and licking when the alert was generated.^a^

| Pruritus activity | | Study period | |
| --- | --- | --- | --- |
| Scratching^b^ | Licking^b^ | First 10-month Study Period  (n = 7191 dogs) | Second 10-month Study Period^c^  (n = 6684 dogs) |
| *Total Number of Alerts, n (%)* | | 113,530 (100) | 93,217 (100) |
| Infrequent | Occasional | 9934 (8.8) | 14,595 (15.7) |
| Infrequent | Elevated | 7974 (7.0) | 11,244 (12.1) |
| Infrequent | Severe | 431 (0.4) | 2868 (3.1) |
| Occasional | Infrequent | 4351 (3.8) | 7050 (7.6) |
| Occasional | Occasional | 32,002 (28.2) | 15,848 (17.0) |
| Occasional | Elevated | 19,772 (17.4) | 10,541 (11.3) |
| Occasional | Severe | 794 (0.7) | 2837 (3.0) |
| Elevated | Infrequent | 1574 (1.4) | 3257 (3.5) |
| Elevated | Occasional | 16,575 (14.6) | 7903 (8.5) |
| Elevated | Elevated | 13,795 (12.2) | 6715 (7.2) |
| Elevated | Severe | 913 (0.8) | 2480 (2.7) |
| Severe | Infrequent | 197 (0.2) | 834 (0.9) |
| Severe | Occasional | 1992 (1.8) | 2382 (2.6) |
| Severe | Elevated | 2746 (2.4) | 2884 (3.1) |
| Severe | Severe | 480 (0.4) | 1779 (1.9) |

^a^Pruritus alerts were retrospectively generated for the entire study period using the Whistle algorithm to extract alert dates. An alert was generated whenever the scratching or licking activity increased by one or more category levels from the previous day (e.g., from infrequent to occasional).

^b^Behavior levels were quantified as infrequent (scratching, 0 to 52 s/day; licking, 0-7 min/day), occasional (53-119 s/day; licking, 7‑19 min/day), elevated (scratching 120-299 s/day; licking, 19-43 min/day), and severe (scratching > 300 s/day; licking, > 43 min/day).

^c^In the second 10-month period, reports on pruritic behaviors were viewable by dog owners and alerts indicating increased pruritic behaviors were sent to dog owners via digital messages.

**Supplementary Table 5.** Summary of medications prescribed by study period.^a^

| Dog Dermatitis  Category^b^ | Number (%) of medications prescribed by time interval (time after pruritus alert) | | | | | Medications prescribed outside of the 4-week window | Total medications prescribed at any time during the study period |
| --- | --- | --- | --- | --- | --- | --- | --- |
|  | 1 week | 2 weeks | 3 weeks | 4 weeks | Sum of weeks 1-4^c^ |  |  |
| **First 10-month study period** | | | | | | | |
| Group 0  (n = 2845) | 221 | 52 | 20 | 5 | 298  (33.3%) | 596  (66.7%) | 894 |
| Group 1  (n = 2495) | 864 | 228 | 75 | 56 | 1223  (39.1%) | 1905  (60.9%) | 3128 |
| Group 2  (n = 1684) | 1398 | 371 | 166 | 67 | 2002  (37.9%) | 3285  (62.1%) | 5287 |
| Group 3  (n = 167) | 512 | 119 | 20 | 27 | 678  (44.6%) | 842  (55.4%) | 1520 |
| Total  (n = 7191) | 2995 | 770 | 281 | 155 | 4201  (38.8%) | 6628  (61.2%) | 10,829 |
| **Second 10-month study period** | | | | | | | |
| Group 0  (n = 3062) | 1244 | 479 | 319 | 173 | 2215  (56.5%) | 1706  (43.5%) | 3921 |
| Group 1  (n = 2025) | 640 | 330 | 163 | 125 | 1258  (53.7%) | 1084  (46.3%) | 2342 |
| Group 2  (n = 1440) | 818 | 387 | 239 | 148 | 1592  (55.7%) | 1267  (44.3%) | 2859 |
| Group 3  (n = 157) | 247 | 76 | 40 | 29 | 392  (52.9%) | 349  (47.1%) | 741 |
| Total  (n = 6684) | 2949 | 1272 | 761 | 475 | 5457  (55.3%) | 4406  (44.7%) | 9863 |

^a^Medications included agents in the following categories: antipruritics, antibiotics, otics, topicals, and nutritionals. Specific agents in each of these categories are listed in the footnotes to main Table 1 in the paper.

^b^Group 0: 0 visits with dermatitis outcome; Group 1: 1 visit with dermatitis outcome; Group 2: 2-5 visits with dermatitis outcome; Group 3: > 6 visits with dermatitis outcome.

^c^28-day period.

**Supplementary Table 6**. Summary of medications prescribed to dogs in Group 0 (the subgroup with no veterinary clinic visits with a dermatitis outcome) by study period. This subgroup included 2845 dogs in the first 10-month period and 3062 dogs in the second 10-month period.

| Medication  Category | Number (%) of medications prescribed by time interval (time after pruritus alert) | | | | | Medications prescribed outside of the 4-week window | Total medications prescribed at any time during the study period |
| --- | --- | --- | --- | --- | --- | --- | --- |
|  | 1 week | 2 weeks | 3 weeks | 4 weeks | Sum of weeks 1-4^a^ |  |  |
| **First 10-month study period** | | | | | | | |
| Antipruritics^b^ | 87 | 17 | 7 | 2 | 113  (33.3%) | 226  (66.7%) | 339 |
| Antibiotics^c^ | 64 | 19 | 7 | 1 | 91  (32.9%) | 186  (67.1%) | 277 |
| Otics^d^ | 85 | 19 | 9 | 0 | 113  (33.3%) | 226  (66.7%) | 339 |
| Topicals^e^ | 9 | 3 | 0 | 1 | 13  (31.7%) | 28  (68.3%) | 41 |
| Nutritionals^f^ | 7 | 2 | 0 | 1 | 10  (32.3%) | 21  (67.7%) | 31 |
| **Second 10-month study period** | | | | | | | |
| Antipruritics^b^ | 581 | 245 | 157 | 84 | 1067  (59.4%) | 728  (40.6%) | 1795 |
| Antibiotics^c^ | 376 | 131 | 91 | 42 | 640  (55.2%) | 519  (44.8%) | 1159 |
| Otics^d^ | 256 | 105 | 77 | 32 | 470  (55.9%) | 371  (44.1%) | 841 |
| Topicals^e^ | 156 | 50 | 31 | 26 | 263  (52.1%) | 242  (47.9%) | 505 |
| Nutritionals^f^ | 27 | 8 | 3 | 3 | 41  (46.6%) | 47  (53.4%) | 88 |

^a^28-day period.

^b^Antipruritic medications included lokivetmab, chlorpheniramine, cyclosporine, dexamethasone, diphenhydramine, hydroxyzine, oclacitinib, prednisone, trimeprazine/prednisone.

^c^Antibiotic medications included amoxicillin, amoxicillin clavulanate, bacitracin/neomycin/polymyxin, cefadroxil, cefazolin, cefovecin, cefpodoxime, clindamycin, doxycycline, enrofloxacin, enrofloxacin/silver sulfadiazine, gentamicin/betamethasone, gentamicin/clotrimazole/mometasone, marbofloxacin, nystatin/neomycin/thiostrepton/triamcinolone acetonide, sulfadimethoxine/ormetoprim, sulfamethoxazole/trimethoprim.

^d^Otic medications included acetic acid/boric acid, acetic acid/hydocortisone, ear cleaner non-medicated, enrofloxacin/silver sulfadiazine, florfenicol/terbinafine/betamethasone acetate, florfenicol/terbinafine/mometasone furoate, fluocinolone/dimethyl sulfoxide, gentamicin/betamethasone, gentamicin/clotrimazole/mometasone, ketoconazole/chlorhexidine/tris-EDTA, tromethamine/disodium EDTA dihydrate.

^e^Topical medications included acetic acid/chlorhexidine/ketoconazole, benzoyl peroxide, benzoyl peroxide/sulfur/salicylic acid, chlorhexidine /climbazole, chlorhexidine/tromethamine/disodium EDTA dihydrate, conditioner non-medicated, hydrocortisone/aluminum acetate, nystatin/neomycin/thiostrepton/triamcinolone acetonide, sulfur/salicylic acid.

^f^Nutritional treatments included dermatology diet and fish oil/omega fatty acids/vitamin E.
